# Reply to: “The Relationship between Eye Movements and Reading Difficulties”, Blythe, Kirkby & Liversedge

**DOI:** 10.3390/brainsci8060099

**Published:** 2018-06-04

**Authors:** John Stein

**Affiliations:** Department Physiology, Anatomy & Genetics, University of Oxford, Oxford OX1 3PT, UK; john.stein@dpag.ox.ac.uk; Tel.: +44-8651-272552

**Keywords:** eye movements, visual magnocellular system, reading, dyslexia

## Abstract

This is my response to the critique by Blythe et al. of my review ‘What is Developmental Dyslexia?’. In this response, I provide greater detail about the evidence supporting the view that faulty eye movement control can cause dyslexics’ visual reading difficulties and that impaired development of the visual magnocellular system may be the underlying cause.

I am grateful to Blythe et al. for giving me the opportunity to develop the themes outlined in a necessarily brief two paragraphs (<5% of the whole) in my review, “What is Developmental Dyslexia?” [1] Their critique boils down to 2 main points, namely that: (1) the review was misleadingly selective in the results discussed; and (2) it did not prove that a visual magnocellular impairment can cause eye movement deficits and that these can subsequently cause reading impairment.

With regard to the selection of the literature discussed, certainly these 2 paragraphs do not provide a meta-analysis of all the studies in this area, which would not have been appropriate for the aims of the review. However, a meta-analysis of all the studies in this area would have demonstrated a consensus that there is no difference between dyslexics and controls in their ‘main sequence’—the consistent relationship between duration, peak velocity and magnitude of saccades, which is usually measured from just one eye. This was pointed out by Blythe et al. This implies that there are no large differences between the dyslexics and good readers in their brainstem output control of the metrics of their eye movements. On the other hand, when dyslexics attempt to read text, it is generally agreed that their higher control of eye movements is abnormal. For example, saccadic accuracy, amplitude, fixation duration, number of regressions [2] and vergence errors (which require binocular recording that has only become readily available in the last 10 years) are all disturbed. This implies that the cortical and cognitive control of their eye movements when reading text is indeed abnormal.

However, whether these abnormal eye movements are a cause or a consequence of their reading disabilities is disputed. Are they entirely the result of the subjects’ difficulties in understanding the text that they are trying to read? If abnormal eye movements are not observed during eye movement tasks that are similar to reading but do not involve decoding text, clearly the eye movement abnormalities seen when reading are likely to be a consequence of their difficulties deciphering the text rather than their cause, as was argued by Rayner [2]. However in 1981, Pavlidis was the first to show that some dyslexics do have abnormal eye control even when fixating on a row of sequentially illuminated LEDs [3]. These 13-year-old children were selected because they had bizarre spelling and tended to reverse their letters. They all showed inaccurate saccades between the lights, unstable fixation on the lights and frequent reversals back to the previously illuminated ones, while the other 12 normal readers showed none of these abnormalities. Although Pavlidis’s results were confirmed by many later authors, who used better eye movement recording techniques [4,5,6,7,8], several other influential authors failed to do so [9,10,11]. The probable reason for this disagreement is that similar to Pavlidis’s, the studies that showed eye control abnormalities may have chosen mainly children with visual reading problems, whereas those that did not show abnormalities chose children with mainly phonological problems whose difficulties may have resulted primarily from auditory processing abnormalities. Nobody claims that all dyslexics have visual processing and eye movement problems; so the choice of subjects is crucial.

Another way of establishing cause and effect is to compare the eye control of dyslexics with younger children whose reading is already at the same level as the dyslexics. These younger children will have had the same reading experience as the older children with dyslexia. Several studies have confirmed that these younger children exhibit better eye control than their older dyslexic counterparts [12,13], implying that their reading advantage for their age was indeed partially due to their superior eye control.

However, the most compelling evidence comes from interventions. Many studies have now confirmed that improving magnocellular function improves eye control, which can significantly improve reading in many dyslexics [13,14,15,16,17]. These three consistent lines of evidence convince me that faulty eye movement control can indeed contribute to many children’s visual reading problems.

On the question of whether the visual magnocellular system is involved in these eye control abnormalities, there is now overwhelming evidence that many children with dyslexia have impaired magnocellular development [18,19]. As it is also generally agreed that this system plays the dominant role in the visual guidance of eye movements [20], its impaired development will clearly compromise rapid and accurate eye control, hence help to cause reading problems. Therefore, there is now strong evidence that impaired visual magnocellular function does indeed contribute to many children’s reading difficulties. Disagreement with this conclusion probably arises from which subjects were studied, as well as the small sample sizes in most of the reports. If the majority of the children studied have visual reading symptoms, their visual magnocellular deficit stands out. However, if they have mainly phonological difficulties and auditory temporal processing weaknesses, any visual magnocellular deficit is difficult to detect.
